# Comparative evaluation of physiological and molecular responses of blackcurrant varieties to powdery mildew infection

**DOI:** 10.3389/fpls.2024.1445839

**Published:** 2024-09-17

**Authors:** Weihua Li, Dong Qin, Ruiqun Ma, Shuxian Li, Lin Wang

**Affiliations:** College of Horticulture and Landscape Architecture, Northeast Agricultural University, Harbin, China

**Keywords:** blackcurrant, powdery mildew, resistance enzyme activity, endogenous hormone, transcriptome

## Abstract

The black currant (*Ribes nigrum* L.), a member of the Saxifragaceae family’s *Ribes* genus, has gained consumer and grower acceptance due to its high nutritional value and economic potential. However, powdery mildew, the primary leaf disease affecting black currants, significantly impacts growers and the industry. Developing varieties highly resistant to powdery mildew is currently considered the most scientifically sound solution. However, the black currant’s physiological and disease resistance mechanisms post-infection by powdery mildew remain understudied, thereby impeding further breeding efforts. Therefore, this study aimed to elucidate the pathogenesis of powdery mildew in various susceptible varieties, post-infection physiological changes, and molecular mechanisms related to powdery mildew. This was achieved through phenotypic observation, physiological data analysis, transcriptomic analysis, and qRT-PCR-mediated gene expression analysis.

## 1 Introduction

Blackcurrant (*Ribes nigrum* L.), a perennial deciduous shrub in the Saxifragaceae family (Weigend, 2007), is one of over 160 Ribes species worldwide, with 59 species found in China. It thrives in cooler climates around the 45°N latitude, such as Northern Europe, North America, Northeast China, and Xinjiang. Blackcurrant is highly nutritious and widely popular, with most varieties containing over 250 mg/g fresh weight (FW) (Rune and Haavard, 2002) of anthocyanin. Mature black currant fruits have vitamin C content ranging from 120-280 mg/100 FWg (Qing et al., 2021). Additionally, blackcurrants are rich in flavonoids like lutein, quercetin, and carsonic acid, which help combat inflammation and neurodegeneration (Tímea et al., 2023). The seeds are high in γ-linolenic acid (Joanna et al., 2021), known for its anti-cardiovascular and anti-cancer properties (Farag Mohammed et al., 2023). However, blackcurrants are vulnerable to powdery mildew infection caused by *Podosphaera mors-uvae*, leading to brittle, wrinkled leaves that eventually die, thus significantly impacting production in China and causing substantial economic losses for growers.

Malondialdehyde accumulation can disrupt plant cell membrane structure and function, causing cell contents to leak and relative conductivity to rise, thereby serving as a membrane damage indicator (QiuFen et al., 2023). Furthermore, the accumulation increases in wild grapes post-powdery mildew infection (Yingqiang et al., 2012). Plants have a complex antioxidant system, comprising non-enzymatic antioxidants like ascorbic acid, flavonoid, and carotenoid, and enzymatic antioxidants such as catalase (CAT) (Jun et al., 2023), phenylalanine ammoniase (PAL) (Shuwu et al., 2021), peroxidase (POD) (Álvaro et al., 2019), polyphenol oxidase (PPO) (Zhijun et al., 2023), and superoxide dismutase (SOD) (Bhonwong et al., 2009). These antioxidants collaboratively regulate reactive oxygen species (ROS) production and scavenging, thereby shielding plant cells from oxidative damage (Ashikhmin et al., 2023). This protective mechanism is further enhanced by endogenous hormones, including (indole-3-acetic acid) IAA, gibberellic acid (GA_3_), abscisic acid (ABA) and salicylic acid (SA), which significantly affect plant resistance post-powdery mildew infection in watermelon (Vivek et al., 2022), barley (Chen et al., 2013), strawberry (Jun et al., 2020a), and grape (Neetu et al., 2021). In previous studies, it was found that the Sph2 gene (Keep, 1977) determined the resistance of Ojebyn to powdery mildew, while the complementary genes M1 and M2 enhanced the powdery mildew resistance in Brodtorp (Rousi, 1966).

## 2 Materials and methods

### 2.1 Plant materials

Based on the blackcurrant mildew infestation over the past two years, 10 blackcurrant varieties were selected as experimental varieties. The mildew resistant varieties were Ojebyn, Brodtrop, TianMi, 16A, and C19. The susceptible varieties included LiangYe, Binhai Minzhu, Jinian, Zhong 15, and Ben Lomond. The experimental plant materials were sourced from the Small Berry Germplasm Resources Nursery at the College of Horticulture and Landscape Architecture, Northeast Agricultural University, Harbin. The sampling occurred from May to August. High temperature and humidity are conducive to the spread of powdery mildew. All plant material was infected in its natural state, from which the causative agent was isolated and identified as *Podosphaera mors-uvae* by the subject. As temperature and humidity increased, powdery mildew infections became more severe, and were divided into five periods: healthy, beginning, middle, flourishing, and terminal infection. Leaves were cut, cleaned with 70% alcohol, placed in freeze-thaw tubes, rapidly frozen in liquid nitrogen, and stored at -80°C. Each group had three biological replicates.

### 2.2 Spray treatments

For the LiangYe varieties in the early stage of infestation, treat powdery mildew by spraying triadimefon (800x dilution) with spray cans in the afternoon during calm, dry weather, ensuring no rain for three hours post-application. Spraying was repeated after 12 days. When spraying drugs, plastic film was used to prevent the drug from affecting the powdery mildew disease of other plants.

### 2.3 Classification of powdery mildew infestation periods

Healthy: The leaf surface appears healthy, without any white powder.

Beginning infestation: White powder is visible on the back of leaves.

Middle infestation: Over 25% of the leaf blade’s back is infested with the powdery mildew pathogen, with small amounts visible on the front.

Flourishing infestation: Both sides of the leaf blade are heavily infested, causing wrinkling due to the pathogen infestation.

Terminal of infestation: Many leaves are crumpled or withered from severe powdery mildew infestation.

Specific levels are illustrated in Figure 7 in the **Appendix**.

### 2.4 Measurement of photosynthetic indexes

The measured values include: maximum photochemical efficiency (Fv/Fm); electron transfer per unit reaction center (ETo/RC); energy absorbed per unit reaction center (ABS/RC); energy captured per unit reaction center (TRo/RC); energy absorbed per unit reaction center (ABS/CSm); photosystem II performance index (PI abs); photosystem II total performance (PI total); and five quantum yield data [phi(po), psi(Eo), delta(Ro), phi(Ro), Phi(Eo)]. Relative chlorophyll content (SPAD) was also measured. Chlorophyll fluorescence data were obtained using a Portable Photosynthetic Efficiency Analyzer (PEA, Hansatech Inc. Co., UK) on a clear, windless morning. Leaves selected for each plant were of similar color and size, with five measurements conducted per plant. Relative chlorophyll content (SPAD value) of upper leaves of black currant plants was determined using a SPAD chlorophyll meter.

### 2.5 MDA and five antioxidant enzyme activity assays

For malondialdehyde determination, leaves were ground with trichloroacetic acid (TCA), centrifuged to collect the supernatant, mixed with thiobarbituric acid (TBA), and boiled in a water bath. Absorbance values were measured at 532 nm and 450 nm (Zhaorong et al., 2023).

For antioxidant enzyme activity measurement, leaves were homogenized in phosphate buffer solution (PBS), centrifuged at 10000 g at 4°C for 20 min (Jinhua et al., 2022), and the supernatant was used. The activity of superoxide dismutase (SOD), polyphenol oxidase (PPO), peroxidase (POD), and catalase (CAT) was determined spectrophotometrically using the nitroblue tetrazolium (NBT) method, guaiacol assay, H_2_O_2_ (Lifeng et al., 2014), and pyrogallic acid method, respectively.

The phenylalanine deaminase (PAL) assay, adapted from Sellamuthu (Sellamuthu et al., 2013) with modifications, involves the following procedure. Begin by weighing 0.5 g of leaves, adding 5 mL of 0.1 mol/L boric acid buffer, and homogenizing by grinding. Centrifuge the mixture at 120,000 g/min at 4°C for 20 minutes to obtain the supernatant, which is then incubated in a 37°C water bath for 30 minutes. To 1 mL of this enzyme solution, add L-phenylalanine (0.02 mol/L) and boric acid buffer. Measure the absorbance at 290 nm.

### 2.6 HPLC determination of endogenous hormones

The leaves were first crushed into a powder using liquid nitrogen, then dissolved in 80% methanol. Next, the mixture was sonicated and macerated at -20°C for 16 h. The leaves were extracted with petroleum ether and the supernatant was decolorized by removing residual color with additional petroleum ether. The decolorized supernatant was then evaporated to dryness, and redissolved in 2 mL of HPLC-grade methanol, filtered through a 0.22 μm membrane, and transferred to a brown injection vial for analysis.

The mobile phase included chromatography-grade methanol (phase A) and an aqueous acetic acid solution at pH 3.6 (phase B). The chromatographic column used was a Waters XBridge C18 5 μm (4.6×250 mm). Diode array detection (DAD) was set at 254 nm for IAA, GA3, and ABA determination, with an elution procedure of 1–12 min (A, 55%; B, 45%). For SA determination, the wavelength was 290 nm, with the elution procedure: 0 min (A, 0%), 0–3 min (A, 28%), 3–6 min (A, 45%), 6–9 min (A, 60%), 9–10 min (A, 45%), and 10–15 min (A, 0%).

### 2.7 Leaves RNA extraction and sequencing

The RNAprep Pure Total RNA Extraction Kit (Tiangen Biotech Co.,Ltd., Beijing, China) was used for polysaccharide and polyphenol-rich plants. For reverse transcription, the TOYOBO ReverTra ACE qPCR RT Master Mix with gDNA Remover Kit was used.

BMK conducted library construction and transcriptome analysis. RNA purity and concentration were measured with a NanoDrop 2000 spectrophotometer, and RNA integrity was assessed using the Agient 2100/LabChip GX Sequencing was performed on an Illumina NovaSeq 6000 platform with a PE150 pattern.

LiangYe was introduced into northeast China from present-day Belarus by Russian expatriates and was given the name LiangYe after domestication, but it disappeared due to severe powdery mildew infection. Therefore, LiangYe was selected as a drug spray treatment and as a variety for transcriptome sequencing.

Transcriptome data were categorized into three groups: LYH (LiangYe healthy), LYI (LiangYe powdery mildew infested), and LYM (LiangYe sprayed with medication control). The transcriptome data has been published at NCBI as https://www.ncbi.nlm.nih.gov/bioproject/PRJNA1142740/.

### 2.8 Quantitative real-time PCR

To validate physiological data, enzyme activity and endogenous hormone-related genes showing \ opposing trends and significant changes in transcriptomic data were screened during infection and recovery compared to controls (**Table 1**). Finally, their relative expression was determined using quantitative reverse transcription polymerase chain reaction (qRT-PCR). The qRT-PCR reaction Mixture system is presented in **Table 2**.

**Table 1:** Primer sequences used for qRT-PCR.

Gene ID	Forward (5’-3’)	Reverse (5’-3’)
DN8590_c0_g3	AAAGTAATGGGCGGGTAT	AGATTCTGGGAAGGTGGA
DN7123_c0_g1	CTAACTTGACTGTCGGTGGA	TCAAAGTGTCCGAGCACC
DN2082_c0_g1-F	CAGGTATTCGATGGGAGG	AAGACAACGGCACGAGAT
DN64_c0_g2	TTAACAATGCTGCCCAGGTA	ACCCAGCCAGAGCCAAAT
DN6918_c1_g1	ATTTAGGATATGTGCGTTGA	AGGGAAGTAATGGAGGGA
DN12960_c1_g3	GCTTCTCCACGACGGGTAT	GCGCTGGTGACGCAACTA
DN4946_c0_g1	GGTTTCGGCAGAGTCAGT	CTCATACCCGTTGTCCAG
DN14115_c1_g1	CAAGGCGGTCGTTGTATC	TTTAAGGGCAGTCCATCC
*actin*	TCAACTATGTTCCCTGGTATTGC	CTCCCTTGGAAATCCACATCTG

**Table 2:** qRT-PCR reaction Mixture system.

Ingredient	volume (μl)
SybrGreen qPCR Master Mix	10
Forward primer	0.8
Reverse primer	0.8
ddH_2_O	6.4
cDNA	2
Total	20

### 2.9 Statistical analysis

Data statistical processing and correlation analysis were performed using Microsoft Excel and SPSS 22.0, while plotting was done with Microsoft Excel and Origin software. RNA-seq data analysis was conducted using BMKCloud.

## 3 Results and analysis

### 3.1 Survey of the timing of the onset of black currant

The experiment documented the onset date of each infection stage for ten black currant varieties. In this study year (2023), black currant mildew was particularly severe in Harbin. Powdery mildew first appeared in late May and naturally subsided by mid-August. The flourishing infection from June to July. Resistant varieties typically experienced each stage later than susceptible ones, resulting in lesser impacts on plant growth. **Table 3** shows that JiNian and Bin Haimingzhu entered the initial infection stage 29 days earlier than Ojebyn, the last variety to do so. The five susceptible varieties reached flourishing infection on June 22, while the five resistant varieties reached flourishing infection at least 14 days later.

**Table 3:** Timetable of 10 blackcurrant varieties entering each infestation period.

Species name	Beginning of infestation	Middle infestation	Flourishing infestation	Terminal of infestation
JiNian	5/21	6/2	6/22	7/7
BinHaimingzhu	5/21	6/2	6/22	7/11
LiangYe	5/22	6/2	6/22	7/10
Zhong15	5/22	6/4	6/22	7/10
Ben Lomond	5/29	6/12	6/22	7/10
C19	5/29	6/22	7/11	7/17
TianMi	5/29	6/23	7/11	7/17
Brodtrop	5/29	6/24	7/6	7/15
16A	6/11	6/23	7/22	8/3
Ojebyn	6/19	6/24	7/6	7/11

### 3.2 Differences in photosynthetic indexes between resistant and susceptible varieties

#### 3.2.1 Differences in SPAD values between resistant and susceptible varieties

**Figure 1:**
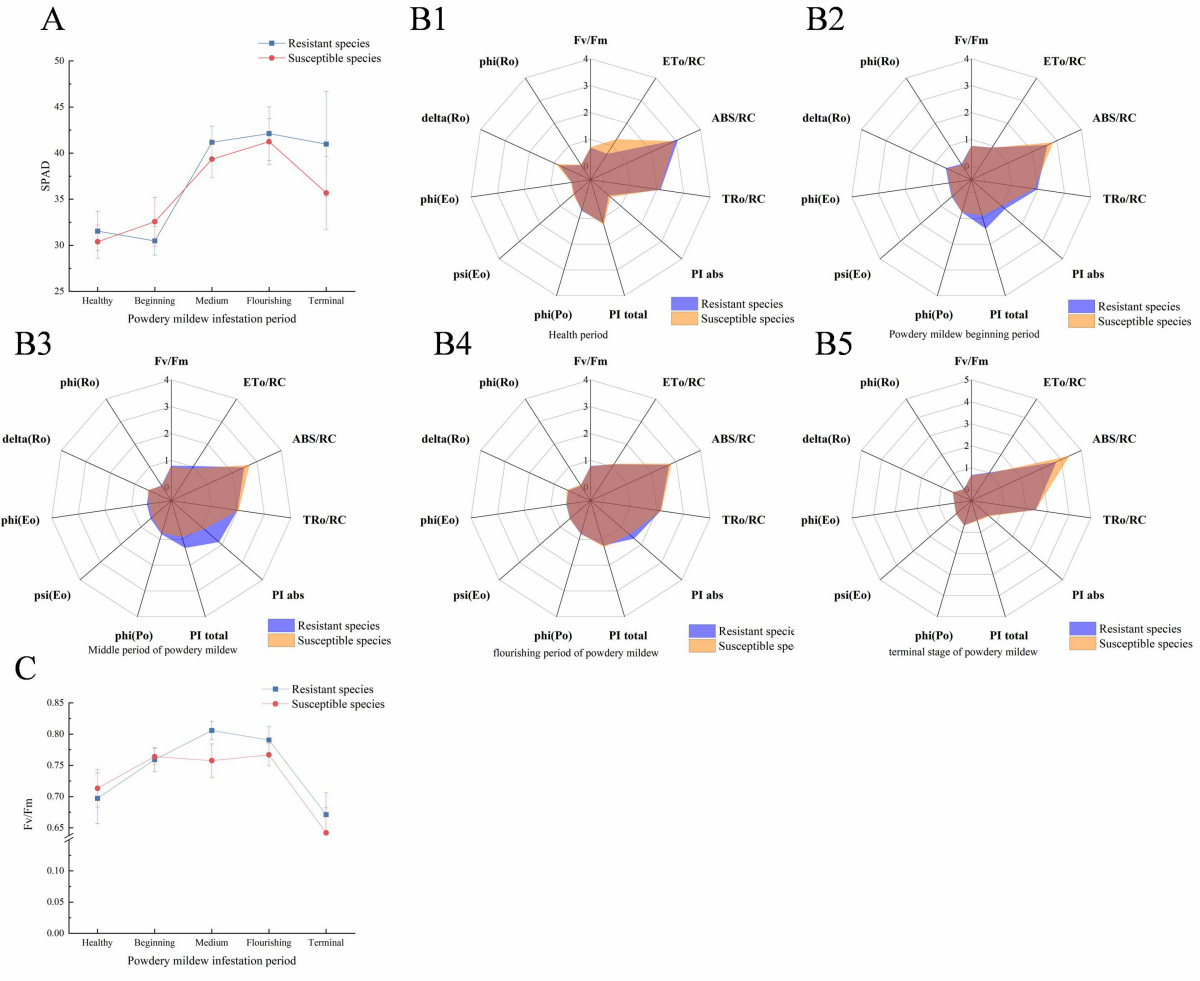
Line graphs and radar plots of changes in photosynthetic indicators. **(A)** Line chart of SPAD value change of susceptible varieties post-powdery mildew infection; **(B1–B5)**. The chlorophyll fluorescence radar map of the healthy, beginning of infestation, middle infestation, flourishing infestation, and terminal of infestation periods, respectively. **(C)** Line chart of Fv/Fm changes after infection of susceptible varieties with powdery mildew. The average of resistant and susceptible varieties in **(A, C)** was calculated from five biological replicates and three technical replicates. The coordinates represent the infection period (horizontal) and its corresponding value (vertical).

Powdery mildew significantly impacted black currant leaf photosynthesis, with the relative chlorophyll content (SPAD) indicating changes in leaf chlorophyll levels. **Figure 1A** shows that the average SPAD value of susceptible varieties initially increased, then decreased, peaking at the flourishing infection stage. Resistant varieties had a higher peak SPAD value (42.12) compared to susceptible ones (41.24). Throughout the infestation, the SPAD content of resistant varieties remained higher, with a notable 14.9% difference at the terminal stage.

#### 3.2.2 Differences in chlorophyll fluorescence between resistant and susceptible varieties

During the healthy period, resistant varieties ABS/RC and TRo/RC exhibited greater resistance compared to susceptible varieties (**Figure 1B1**). However, ETo/RC demonstrated significant resistance against susceptible varieties, indicating that the leaf efficiency of susceptible varieties exceeded that of resistant ones during this phase. At the onset of infestation, resistant varieties, except ABS/RC, showed higher leaf work efficiency than susceptible varieties (**Figure 1B2**), suggesting a stronger leaf work efficiency in resistant varieties. At the flourishing of infestation, the differences in the three parameters (PI abs, PI total, and ETo/RC) between resistant and susceptible varieties increased further (**Figure 1B4**). During this period, only PI abs and Fv/Fm were higher in resistant varieties, while most other parameters were similar to those of susceptible varieties. This indicates significant damage to the physiological function of resistant leaves during flourishing infestation. By the terminal stage of infestation, all parameters of resistant varieties, except ABS/RC, significantly decreased and aligned with those of susceptible varieties (**Figure 1B5**). This suggests severe damage to the photosynthetic system in both resistant and susceptible varieties post-powdery mildew infestation. The maximum photochemical efficiency (Fv/Fm) reflects the photosynthetic efficiency of leaves and thus the degree of leaf damage. As shown in **Figure 1C**, resistant and susceptible varieties exhibited the lowest values at the terminal of infestation, only 82.7% and 83.7% of the peak value. These results indicate severely reduced photosynthetic efficiency and significant damage to the photosynthetic system in black currant leaves at the infestation’s terminal stage. This further confirms the irreversible effects of powdery mildew on black currant leaves at this stage. **Figures 1B1–B5** shows that ABS/RC parameters were higher in susceptible varieties than in resistant ones during all periods, except the healthy period, and increased sharply at the terminal infestation. This suggests a positive correlation between ABS/RC and the severity of powdery mildew infection. Although black currant resistant varieties can delay powdery mildew infection more effectively than susceptible varieties, they cannot completely eliminate or resist the pathogen.

### 3.3 Differences in resistance enzyme activity between resistant and susceptible varieties

**Figure 2:**
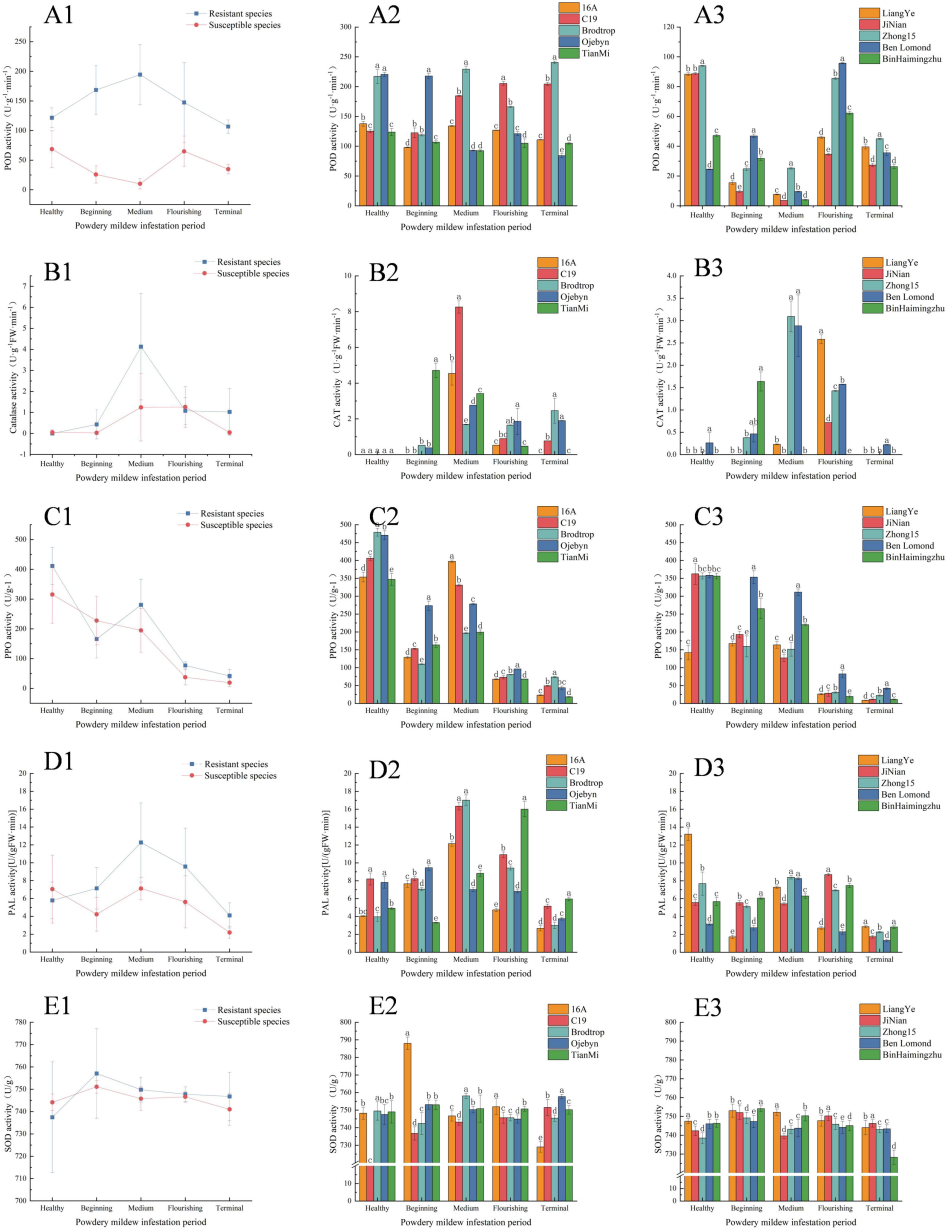
Line and bar graphs of changes in resistance enzyme activity. The columns labelled 1, 2, and 3 in **Figures 1**, **2** and **3**, respectively, represent line graphs of the mean activity of resistant and susceptible varieties, histograms of the activity of resistant varieties, and histograms of the activity of susceptible varieties. **(A–E)** represent the POD, CAT, PPO, PAL, and SOD activities, respectively. The horizontal and vertical coordinates indicate the infestation period and enzyme activity values, respectively.

As shown in **Figure 2** (B1, D1, F1), after infestation with powdery mildew, the CAT, PAL, and SOD activities initially increased and then decreased. In contrast, PPO activity consistently decreased (**Figure 2C1**). The POD activity of resistant and susceptible varieties exhibited different trends: resistant varieties showed an increase followed by a decline, while susceptible varieties decreased, then increased, and finally decreased again (**Figure 2A1**).

During the flourishing infestation period, the POD activity of resistant varieties reached 241.266 U·g^-1^·min^-1^, but declined to 122.632 U·g^-1^·min^-1^ by the terminal infestation (**Figures 2A2, A3**). In contrast, susceptible varieties initially decreased to 9.775 U·g^-1^·min^-1^, increased to 82.162 U·g^-1^·min^-1^ during flourishing infestation, and then rapidly decreased. Throughout the infestation, POD activity in resistant varieties remained higher than in susceptible varieties, peaking at 2.9 times higher. **Figures 2B2, B3** showed that CAT activity in resistant varieties peaked at 4.127 U·g^-1^FW·min^-1^during middle infestation and 1.259 U·g^-1^FW·min^-1^ during flourishing infestation, which was 3.28 times higher than in susceptible varieties. However, after middle infestation, CAT activity decreased in both resistant and susceptible varieties, possibly due to organellar damage. By the terminal infestation, CAT activity of resistant and susceptible varieties had declined to 1.024 U·g^-1^FW·min^-1^ and 0.044 U·g^-1^FW·min^-1^, respectively. As shown in **Figures 2C2, C3**, the peak PPO activity of resistant varieties occurred during the healthy period, with values of 411.141 U/g and 315.172 U/g, which were 30.45% higher than those of susceptible varieties. However, both resistant and susceptible varieties exhibited an overall decreasing trend, with the lowest values of 41.653 U/g and 19.169 U/g, respectively, by the terminal infestation. Throughout the infestation, PPO activity in resistant varieties was consistently 1.17 times higher than in susceptible varieties, except at the beginning. **Figures 2D2, D3** illustrates the trend of PAL activity, showing an increase and subsequent decrease in resistant varieties, whereas susceptible varieties first decreased, then increased, and decreased again. The peak PAL activities for resistant and susceptible varieties occurred during the middle of the infestation, with values of 12.267 U·g^-1^FW·min^-1^ and 7.122 U·g^-1^FW·min^-1^, respectively. Notably, during the healthy period, PAL activity in susceptible varieties (7.05 U·g^-1^FW·min^-1^) surpassed that in resistant varieties (5.785 U·g^-1^FW·min^-1^) However, except at the beginning of the infestation, PAL activity in resistant varieties was consistently higher than in susceptible varieties. **Figures 2E2, E3** shows that SOD activities in both resistant and susceptible varieties remained high during the infestation period. The average peak activities for resistant and susceptible varieties were observed at the beginning of the infestation (757.035 U/g and 751.116 U/g), indicating a rapid response. Overall, mean SOD activity in resistant varieties was higher than in susceptible varieties, except during the healthy period, when susceptible varieties exhibited greater average SOD activity.

### 3.4 Determination of endogenous hormone content using HPLC

**Figure 3:**
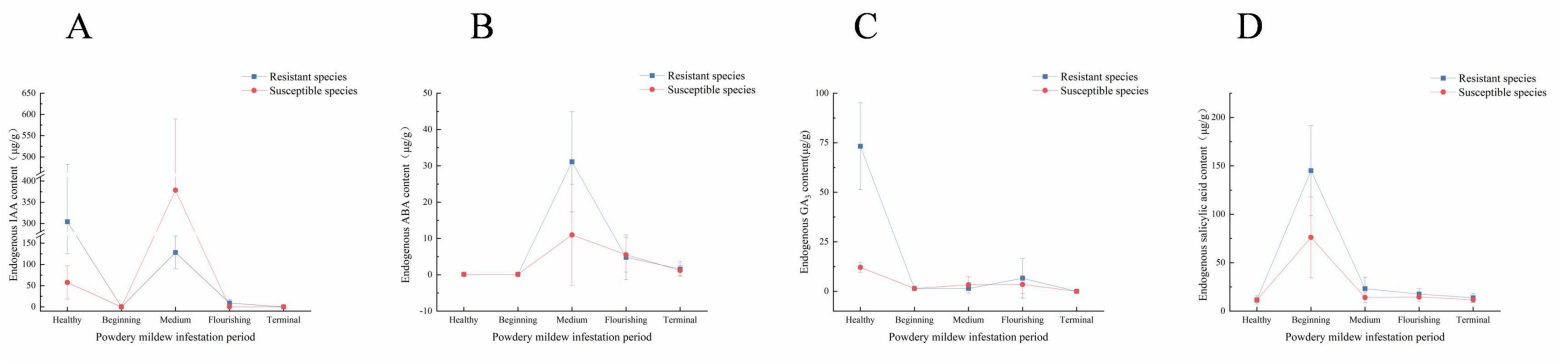
Line graph of endogenous hormone changes. Change of endogenous hormone content post-powdery mildew infection. The horizontal and vertical coordinates indicate the onset period and the endogenous hormone content, respectively. **(A)** Changes of endogenous IAA content; **(B)** Changes of endogenous ABA content; **(C)** Changes of endogenous GA_3_ content; **(D)** Changes of endogenous SA content.

As shown in **Figure 3**, after being infested with powdery mildew, the resistant black currant varieties exhibited fluctuating levels of endogenous IAA, ABA, and SA, with an initial decrease, followed by an increase, and another decrease. The endogenous GA_3_ levels consistently decreased. ABA, GA_3_, and SA were found to positively regulate powdery mildew resistance, whereas IAA negatively affected resistance.

As shown in **Figure 3A**, endogenous IAA peaked in resistant varieties during the healthy period and in susceptible varieties at the midpoint of infestation. In resistant varieties, IAA levels were higher during both the healthy period and the flourishing of infestation, while in susceptible varieties, levels were higher during the middle of infestation. During the healthy period, IAA primarily influenced plant growth and development, which may explain the faster growth observed in resistant varieties compared to susceptible ones. However, post-infestation, increased IAA levels were inversely related to resistance, suggesting that elevated IAA made the plant more susceptible to further infestation. The low endogenous IAA levels throughout the infestation period might have resulted from the pathogen’s destruction of the IAA synthesis pathway. As depicted in **Figure 3B**, endogenous ABA levels in resistant and susceptible varieties peaked at 31.122 μg/g and 10.984 μg/g, during middle infestation. During flourishing infestation, susceptible varieties exhibited higher levels of ABA than resistant varieties, whereas at other times, levels were either lower or equal. This indicates that ABA responds to powdery mildew later and accumulates significantly during middle infestation. Endogenous ABA levels were very low during healthy and early infested periods. As shown in **Figure 3C**, following powdery mildew infestation, endogenous GA_3_ levels in resistant varieties decreased and eventually reached 0 μg/g. During the healthy period, resistant varieties had much higher GA3 levels (73.22 μg/g) compared to susceptible varieties (12.94 μg/g), a difference of 5.66 times. Endogenous GA_3_ levels were lower in the early and late stages of infestation. As shown in **Figure 3D**, endogenous SA levels in resistant varieties peaked early during infestation, accumulating rapidly to combat the pathogen. The peak values were 145.076 μg/g and 76.105 μg/g for resistant and susceptible varieties, respectively, a difference of 1.91 times. The mean endogenous SA levels in resistant varieties were higher than in susceptible varieties at all times except the beginning of infestation, where they remained low. This suggests that SA primarily functions as a signaling molecule to activate the plant’s defense system.

### 3.5 Transcriptome analysis of black currant leaves

**Figure 4:**
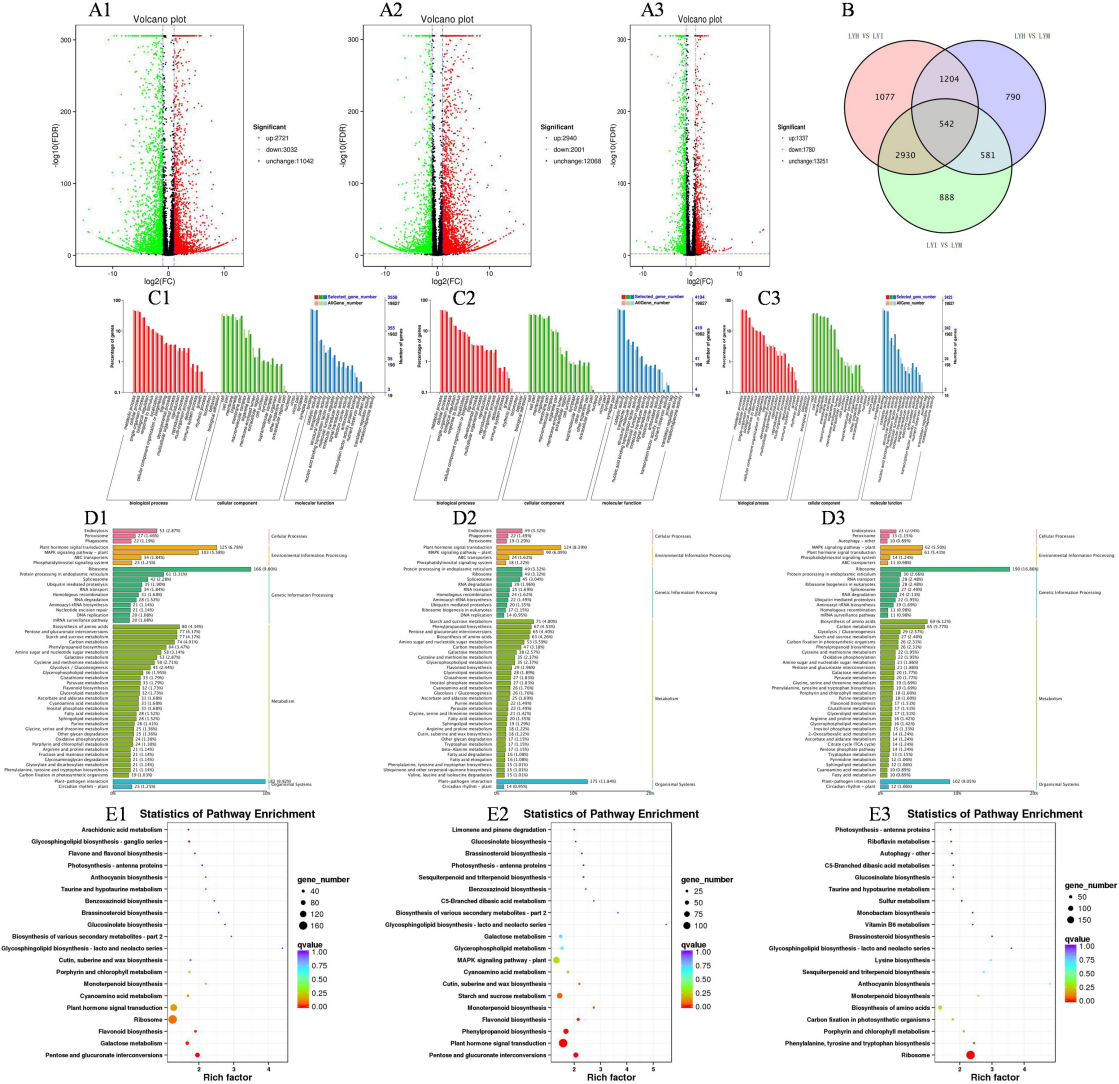
Volcano, bar and bubble plots of transcriptome data. **(A1, C1, D1, E1)** represent LYH vs LYI group; **(A2, C2, D2, E2)** represents LYI vs LYM group respectively; **(A3, C3, D3, E3)** represents LYH vs LYM group respectively. **(A1, A2, A3)** The volcano indicates the DEGs. **(B)** The Venn diagram of LYH, LYI, LYM. **(C1, C2, C3)** GO analysis of DEGs classified as biological, cellular, or molecular functions. **(D1, D2, D3)** Analysis of DEGs classified as cellular processes, environmental information processing, metabolism and organismal systems. **(E1, E2, E3)** Enrichment analysis of DEGs pathway. The X and Y-axis represent enrichment factor and pathway names, respectively. A colored bubble represents q-value, and Rich Factor refers to the value of enrichment factor, which is the quotient of foreground value (the number of DEGs).

Nine samples underwent transcriptome sequencing, producing 57.80 GB of clean reads post-quality control. Each sample yielded 5.99 GB of clean data, with a base percentage between 92.74% and 94.01%. These results meet the requirements for subsequent data assembly and analysis. A total of 45,918 Unigenes were assembled, with an N50 coefficient of 1946, aligning with Illumina sequencing expectations. The group transfer integrity also meets further analysis requirements. Using BLAST with an E-value ≤ 1e-5 and HMMER with an E-value ≤ 1e-10, 24,579 annotated Unigenes were identified. As shown in **Figures 4A1–A3**, the LiangYe LYH group of black currant exhibited 2,721 up-regulated and 3,021 down-regulated differentially expressed genes (DEGs) compared to the LYI group, with more down-regulated DEGs. The LiangYe LYI group had 2,940 up-regulated and 2,001 down-regulated DEGs compared to the LYM group, with more up-regulated DEGs. The LiangYe LYH group had 1,337 up-regulated and 1,780 down-regulated DEGs compared to the LYM group, with more down-regulated DEGs. The number of up- and down-regulated DEGs in LYH vs LYM group was significantly less than in the other two groups. From the Venn diagram (**Figure 4B**), it is observable that the three groups share 542 DEGs. It can be hypothesized that these DEGs are not strongly correlated with powdery mildew infestation and spray drug treatment, and might be closely related to the growth, development, and self-regulation of the black currant. As shown in **Figures 4C1–C3**, the GO enrichment results for both LYI vs LYH, LYI vs LYM groups, and LYH vs LYM groups were similar, with 55 subcategories enriched across the three main categories: biological process, cellular component, and molecular function. In the biological process category, key subcategories included metabolic process, cellular process, single-organism process, biological regulation, and response to stimulus. The cellular component category primarily included cell, cell part, membrane, organelle, and macromolecular complex. The molecular function category focused on binding, catalytic activity, and transporter activity.

As shown in **Figure 4D1**, the LYH vs LYI group exhibited the highest number of enriched Unigenes in cellular processes, specifically in endocytosis, accounting for 2.87% of all Unigenes and including 53 DEGs). In environmental signaling, plant hormone signal transduction had the most enriched Unigenes, constituting 6.78% of all unigenes and 125 DEGs. The MAPK signaling pathway-Plant followed, with 5.58% of all Unigenes and 103 DEGs. For genetic information processing, the ribosome pathway was the most enriched, representing 9.00% of all unigenes and 166 DEGs. Among metabolites, the most enriched pathway was the biosynthesis of amino acids, accounting for 4.34% of all unigenes and 80 DEGs. This was followed by pentose and glucuronate interconversions, and starch and sucrose metabolism, both at 4.17% of all unigenes. The top five pathways also included carbon metabolism and phenylpropanoid biosynthesis. In biological systems, the plant-pathogen interaction pathway was the most enriched, comprising 9.92% of all Unigenes and 188 DEGs.

As shown in **Figure 4D2**, endocytosis was the most enriched unigene in the LYI vs LYM group, representing 3.32% of all Unigenes and 49 DEGs, which is lower compared to the LYH vs LYI group. In environmental signal processing, plant hormone signal transduction was the most enriched unigene, accounting for 8.39% of all Unigenes and 124 DEGs. For genetic information processing, ribosome and protein processing in the endoplasmic reticulum were the most enriched, representing 3.32% of all unigenes and 49 DEGs, which were significantly lower than the LYH vs LYI group. In terms of metabolite-enriched substances, starch and sucrose metabolism was the most enriched, accounting for 4.80% of all unigenes and 71 DEGs, lower than the LYI vs LYM group. This was followed by phenylpropanoid biosynthesis, accounting for 4.53% of all unigenes and 67 DEGs, which was higher than the LYI vs LYM group. Other enriched pathways included pentose and glucuronate interconversions, biosynthesis of amino acids, amino sugar and nucleotide sugar metabolism, and carbon metabolism. In biological systems, plant-pathogen interaction was the most enriched, representing 11.84% of all unigenes and 175 DEGs, which is lower than the LYH vs LYI group.

It is imperative to direct particular attention to the ribosome depicted in **Figures 4D3, E3**, in addition to the elevated Rich factor of Phenylalanine tyrosine and tryptophan biosynthesis and porphyrin and chlorophyII metabolism.

After conducting KEGG enrichment analysis on the differentially expressed genes between the LYI and LYH groups and between the LYI and LYM groups, we visualized the top 20 significantly enriched KEGG pathways (**Figures 4E1, E2**). Among these, 13 pathways were common to both comparisons: glucosinolate biosynthesis, brassinosteroid biosynthesis, photosynthesis-antenna proteins, benzoxazinoid biosynthesis, secondary metabolite biosynthesis, glycosphingolipid biosynthesis (lacto and neolacto series), galactose metabolism, cyanoamino acid metabolism, cutin, suberine, and wax biosynthesis, monoterpenoid biosynthesis, flavonoid biosynthesis, plant hormone signal transduction, and pentose and glucuronate interconversions. Among these, monoterpenoid biosynthesis, flavonoid biosynthesis, plant hormone signal transduction, and pentose and glucuronate interconversions exhibited higher levels of enrichment.

### 3.6 Analysis of signal pathways related to LiangYe powdery mildew in blackcurrant varieties

#### 3.6.1 Monoterpenoid biosynthetic pathway

**Figure 5:**
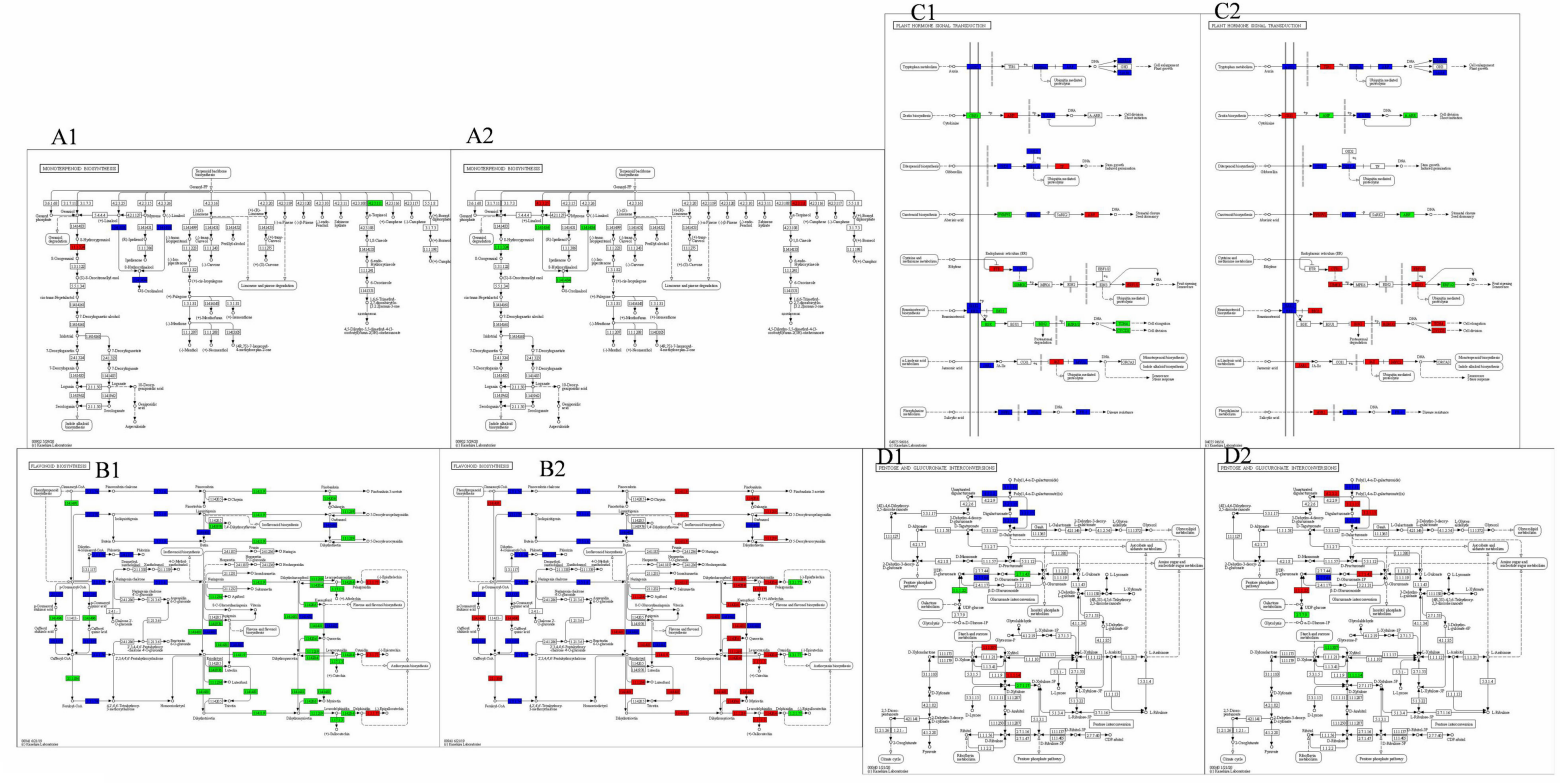
Diagram of the synthetic pathway shared by LYH vs LYI and LYI vs LYM. Red and green indicate up and down regulated expression, respectively, while blue indicates up-down regulated expression. **(A1)** Pathway map of Group LYH vs LYI Monoterpenoid biosynthetic pathway, **(A2)** Pathway map of Group LYI vs LYM flavonoid biosynthesis pathway. **(B1)** Pathway map of Group LYH vs LYI Flavonoid biosynthesis pathway, **(B2)** Pathway map of Group LYI vs LYM flavonoid biosynthesis pathway. **(C1)** Pathway map of Group LYH vs LYI Plant hormone signaling pathways, **(C2)** Pathway map of Group LYI vs LYM Plant hormone signaling pathways. **(D1)** Pathway map of Group LYH vs LYI Conversion pathway of pentose and hexuronic acid, **(D2)** Pathway map of Group LYI vs LYM Conversion pathway of pentose and hexuronic acid.

During both powdery mildew infestation and control, the monoterpene biosynthesis pathway exhibited a higher concentration of differentially regulated genes (**Figures 5A1, A2**). Specifically, 8-hydroxygeraniol dehydrogenase and (E)-8-carboxylinalool synthase genes were up-regulated by 5.13- and 3.06-fold in FPKM values, respectively, while alpha-terpineol synthase genes were down-regulated by 2.33-fold.

Upon artificial spraying of powdery mildew for control, alpha-terpineol synthase genes were up-regulated, contrasting with the down-regulation of 8-hydroxygeraniol dehydrogenase genes. Although (3S)-linalool synthase-related genes showed no significant change during infestation, they were up-regulated during control, albeit with low FPKM values. This suggests a positive correlation between the up-regulation of 8-hydroxygeraniol dehydrogenase and (E)-8-carboxylinalool synthase genes and powdery mildew severity, as their expression decreased post-control. Similarly, alpha-terpineol synthase genes showed a positive correlation, with up-regulation during infestation and decrease post-control. Conversely, alpha-terpineol synthase-related genes exhibited a negative correlation, with down-regulation during infestation and up-regulation post-control.

#### 3.6.2 Flavonoid biosynthesis pathway

The flavonoid biosynthesis pathway also exhibited significant gene expression changes during powdery mildew infestation and control (**Figures 5B1, B2**). Specifically, anthocyanidin reductase-related genes were up-regulated during infestation, whereas 11 related genes were down-regulated. These included trans-cinnamate 4-monooxygenase, caffeoyl-CoA O-methyltransferase, 5-O-(4-coumaroyl)-D-quinate 3’-monooxygenase, flavonol synthase, bifunctional dihydroflavonol 4-reductase/flavanone 4-reductase, flavone synthase II, flavonoid 3’,5’-hydroxylase, anthocyanidin synthase, leucoanthocyanidin reductase, chalcone synthase, and flavonoid 3’-monooxygenase. Additionally, phlorizin synthase-related genes exhibited both up- and down-regulation.

The regulation of genes following powdery mildew spraying for control exhibited an opposite trend to that observed during infestation, except for flavone synthase II. Down-regulated genes during control included those related to anthocyanidin reductase. Conversely, eight genes were up-regulated, including trans-cinnamate 4-monooxygenase, caffeoyl-CoA O-methyltransferase, 5-O-(4-coumaroyl)-D-quinate 3’-monooxygenase, flavonol synthase, bifunctional dihydroflavonol 4-reductase/flavanone 4-reductase, flavonoid 3’,5’-hydroxylase, anthocyanidin synthase, and leucoanthocyanidin reductase. Additionally, nine genes, such as chalcone synthase, chalcone isomerase, flavonoid 3’-monooxygenase, phlorizin synthase, and shikimate O-hydroxycinnamoyltransferase, showed differential regulation. Notably, these five genes exhibited opposite trends during infestation and control.

#### 3.6.3 Plant hormone signaling pathways

**Figures 5C1, C2** illustrates the high number of genes involved in phytohormone signaling pathways during powdery mildew infestation and control. Specifically, six genes were up-regulated during infestation, including histidine-containing phosphotransfer protein, phytochrome-interacting factor 3, ABA responsive element binding factor, ethylene receptor, ethylene-responsive transcription factor 1, and jasmonate ZIM domain-containing protein. Conversely, eight genes were down-regulated, namely abscisic acid receptor PYR/PYL family, mitogen-activated protein kinase kinase 4/5, BRI1 kinase inhibitor 1, BR-signaling kinase, protein brassinosteroid insensitive 2, brassinosteroid resistant 1/2, xyloglucan:xyloglucosyl transferase TCH4, and cyclin D3. Notably, up-regulated genes outnumbered down-regulated ones, totaling 17 related genes. Among these, pathogenesis-related protein 1 showed a significant increase in FPKM value, with a 35.83-fold increase.

After controlling powdery mildew growth through spraying, 13 genes were up-regulated. Notably, brassinosteroid resistant 1/2 and cyclin D3-related genes exhibited significant increases in FPKM values, changing by 5.74-fold and 5.45-fold, respectively. Conversely nine genes were down-regulated, with the FPKM value of DN2265_c0_g2 showing the most substantial decrease, from 15.65 to 0. This change is closely associated with powdery mildew control. Overall, the control process revealed a higher number of up-regulated genes (12) compared to the infestation process, which also had 12 down-regulated genes.

#### 3.6.4 Conversion pathway of pentose and hexuronic acid

Following powdery mildew infestation, only genes related to L-iditol 2-dehydrogenase showed increased expression (**Figures 5D1, D2**). Three other related genes, glucuronokinase, UDP-glucose 6-dehydrogenase, and xylulokinase, were down-regulated. Five additional genes, including pectinesterase, pectate lyase, polygalacturonase, galacturan 1,4-alpha-galacturonidase, and UDP-sugar pyrophosphorylase, exhibited either up- or down-regulation.

In total, four genes were up-regulated, with the pectate lyase-related gene (DN8_c0_g1) showing the highest increase in expression at 41.64-fold. Notably, this gene also decreased by 7.25-fold during infestation, indicating a significant response to powdery mildew. Three genes, L-iditol 2-dehydrogenase, UTP-glucose-1-phosphate uridylyltransferase, and D-xylose reductase, were down-regulated, albeit with minimal expression changes. Dehydrogenase-related genes increased during infestation, displaying varying trends between infested and control samples. Additionally, three other genes, pectinesterase, galacturan 1,4-alpha-galacturonidase, and UDP-sugar pyrophosphorylase, exhibited either up- or down-regulation, with the pectinesterase-related gene (DN13145_c0_g1) showing the highest increase at 43.19-fold.

### 3.7 qRT-PCR analysis

**Figure 6:**
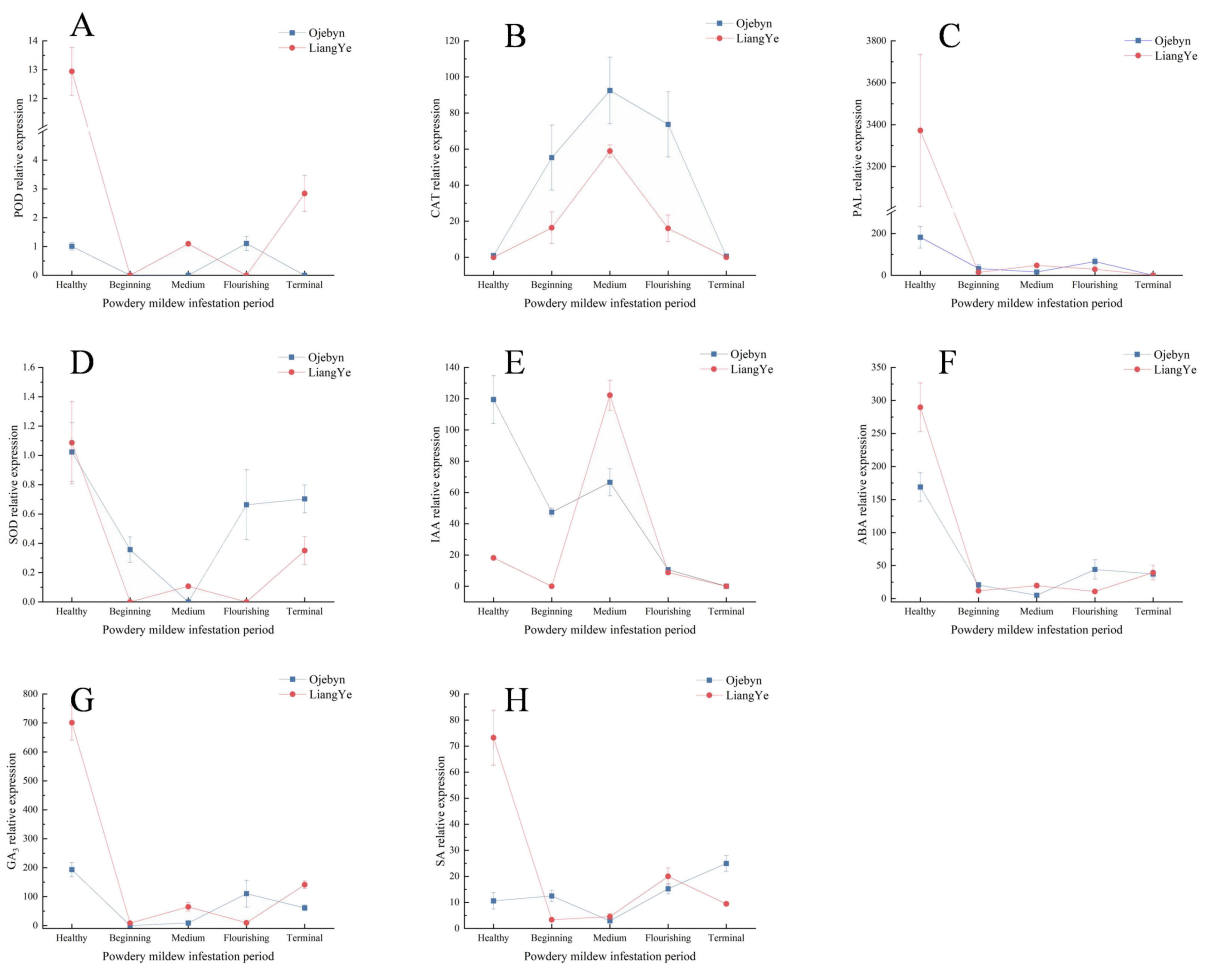
Changes in relative expression of genes associated with physiological data. The horizontal and vertical axes represents the infection duration, and the relative gene expression level, respectively. **(A)** Changes of POD-related gene DN7123_c0_g1 expression; **(B)** Changes of CAT-related gene DN8590_c0_g3 expression; **(C)** Changes of PAL-related gene DN2082_c0_g1 expression; **(D)** Changes of SOD-related gene DN64_c0_g2 expression; **(E)** Changes of IAA-related gene DN4946_c0_g1 expression; **(F)** Changes of ABA-related gene DN12960_c1_g3 expression; **(G)** Changes of GA_3_-related gene DN14115_c1_g1 expression; **(H)** Changes of SA-related gene DN6918_c1_g1 expression.

Eight genes related to physiological indicators were individually screened, and their relative expressions were determined. In **Figures 6A, C, F–H**, the relative expressions of POD, PAL, ABA, GA_3_, and SA generally decreased, peaking during the healthy period. Notably, these peak relative expressions were higher in the susceptible variety, LiangYe, than in Ojebyn. This disparity may stem from powdery mildew presence during the healthy period, prompting earlier regulation and higher expression of these indicator-related genes in Ojebyn. The relative expressions of SOD-related genes (**Figure 6D**) decreased then increased, peaking during the healthy period in both varieties (1.02 and 1.09, respectively). Similarly, the relative expressions of CAT and IAA-related genes (**Figures 6B, E**) decreased, with Ojebyn exhibiting 1.57 times higher expression than LiangYe, peaking middle infestation period (92.48 and 58.89, respectively). The relative expression of IAA-related genes in Ojebyn decreased post-infestation, whereas in LiangYe, it increased before decreasing, peaking at 122.21 middle infestation. Overall, the relative expressions of CAT and IAA genes aligned with their respective physiological data, with both Ojebyn and LiangYe peaking middle infestation. However, Ojebyn’s peak expression of CAT was 1.57 times higher than in LiangYe. Post-infestation, the relative expression of IAA genes decreased in Ojebyn, while in LiangYe, it increased before decreasing, with a peak of 122.21 middle infestation.

**Figure 7:**
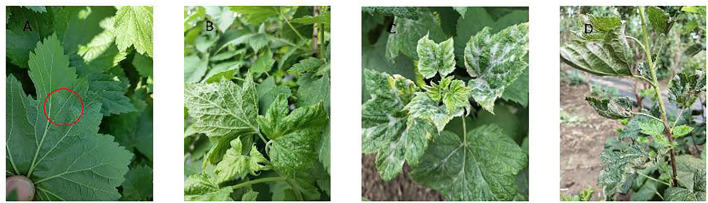
Photographs of various periods of powdery mildew infestation of blackcurrant. **(A)** Beginning of infestation; **(B)** Middle infestation; **(C)** Flourishing of infestation; **(D)** Terminal of infestation.

As shown in **Figures 6A, C, D, F–H**, the peaks of POD, PAL, SOD, ABA, GA_3_, and SA occurred during the healthy period, indicating an early response of related genes to powdery mildew infestation. The relative expression of CAT-related genes gradually increased from this period to the medium stage of infestation. Throughout, the relative expression of Ojebyn exceeded that of LiangYe, suggesting that this gene positively regulates CAT activity and enhances resistance to powdery mildew. However, post-medium infestation, the expression of CAT-related genes decreased, signaling a weakened resistance capacity. Furthermore, while endogenous IAA promotes plant growth during the healthy period, high IAA concentrations post-infestation may not aid in resistance. Notably, resistant varieties enhanced powdery mildew resistance by reducing endogenous IAA levels.

## 4 Discussion

Plant disease resistance arises from complex physiological, biochemical, and molecular mechanisms. Understanding the interaction mechanisms of powdery mildew resistance will provide a foundation for effectively utilizing powdery mildew-resistant black currant materials to develop new, disease-resistant, high-quality varieties.

### 4.1 Effect of powdery mildew of blackcurrant on photosynthetic indices

SPAD values are commonly used to assess biotic stress in plants due to their simplicity and rapid measurement. Studies have shown that tobacco (Shengnan et al., 2022) plants exhibit a swift decrease in SPAD values following powdery mildew infestation. As shown in **Figure 1A**, the SPAD of black currant leaves infested with powdery mildew initially increased and then decreased in both resistant and susceptible varieties. However, throughout the experiment, the SPAD values of resistant varieties remained consistently higher than those of susceptible varieties. This pattern mirrors observations in apple leaves (Xiaomin et al., 2019) infested with powdery mildew, where chlorophyll content initially increases due to the plant’s self-regulation but then declines as the infestation intensifies. The decline is more significant in susceptible varieties due to their weaker regulatory capacity compared to resistant varieties.

### 4.2 Effect of black currant powdery mildew on resistance enzyme activity and MDA

POD, CAT, PPO, PAL, and SOD are crucial for plant resistance to biotic stress (Khodadadi et al., 2020) (Singh et al., 2020). These enzymes scavenge reactive oxygen radicals (Feng et al., 2023), inhibit membrane lipid peroxidation, enhance reactive oxygen metabolism, produce quinones to counteract pathogenic bacterial infestations (Junhong et al., 2014), and directly scavenge oxygen radicals. In tobacco plants inoculated with powdery mildew, POD activity initially increased and then decreased. The resistant varieties exhibited higher POD activity than susceptible varieties (LanJun and Degang, 2023). Similarly, in tobacco plants infested with powdery mildew (Zhijun et al., 2023), CAT activity was greater in resistant varieties compared to susceptible ones. In chili pepper (*Capsicum annuum* L.) plants (Hussein et al., 2023), PPO activity increased after powdery mildew infestation, with probiotics further enhancing PPO activity to a higher peak than the control. This pattern was also observed in grapes (Shimizu and Mazzafera, 2007). In pumpkin seedlings (Bihua et al., 2020), PAL activity rose and then fell following powdery mildew infestation, with PAL positively regulating resistance. Gerbera (Minerva and Kumar, 2019) and grape plants (Zhen et al., 2023) also displayed an increasing and then decreasing trend in SOD activity after infestation, with resistant varieties showing stronger SOD activity than susceptible ones.

**Figure 2** illustrates that all enzymes, except PPO, initially increased and then decreased after powdery mildew infestation. However, in black currant leaves, PPO activity decreased post-infestation, which contrasts with most research findings. Conversely, PPO activity in pumpkin plants showed an overall decrease after infestation, consistent with this experiment’s results. This discrepancy suggests that PPO response mechanisms may vary among plant species against different types of powdery mildew. Despite the varying trends in the five resistance enzyme activities measured in this experiment, resistant varieties consistently exhibited higher enzyme levels than susceptible ones, indicating these enzymes positively regulate powdery mildew resistance.

### 4.3 Effect of powdery mildew of blackcurrant on endogenous hormones

In recent years, some studies have further subdivided endogenous hormones from metabolomics, terming them “endogenous hormone genomics” (Ondřej and Petr, 2023), highlighting their significance. Elevated levels of endogenous IAA or enhanced signaling of its transduction promote plant pathogen infestation (Kunkel and Haeper, 2018). Endogenous IAA has been found to negatively regulate powdery mildew resistance in both wild melon (Ondřej and Petr, 2023) and *Arabidopsis thaliana* (Wang et al., 2007) infested with fungal diseases. Additionally, IAA has been shown to antagonize SA (Yue et al., 2023), reducing powdery mildew resistance. However, another perspective suggests that endogenous IAA growth does not directly oppose the SA-mediated defense system, with SA levels remaining largely unaffected by overexpression of the IAA synthesis gene (Andrew et al., 2013). Rute suggested that the combination of SA and IAA responds more quickly to powdery mildew pathogen *E. necator*, with IAA serving as an early marker (Rute et al., 2022). As shown in **Figure 3**, endogenous IAA and SA contents are negatively correlated in resistant varieties and positively correlated in susceptible varieties. The endogenous IAA content in resistant varieties is much higher than in susceptible varieties during the healthy period and decreased rapidly after infestation. However, in susceptible varieties, endogenous IAA levels were significantly higher than in resistant varieties during the middle infested period. This suggests that while higher endogenous IAA content benefits plant growth and development during healthy periods, it increased significantly after powdery mildew infestation in susceptible varieties, indicating a negative correlation between endogenous IAA content and disease resistance. Additionally, the relative expression of endogenous IAA-related genes (DN4946_c0_g1) in resistant varieties was high in the early stages but rapidly declined after powdery mildew infestation. Conversely, in susceptible varieties, the expression of DN4946_c0_g1 (**Figure 6E**) peaked in the middle stage of infestation, facilitating the spread of powdery mildew pathogens. This demonstrates that endogenous IAA’s role in responding to powdery mildew in blackcurrant varies across different time periods.

Previous studies indicated that susceptible grape varieties maintain high endogenous ABA levels, whereas tolerant varieties exhibited low levels, suggesting that ABA negatively regulates plant resistance to powdery mildew (Rute et al., 2022). ABA regulates stomatal opening, facilitating fungal invasion and enhancing pathogenic bacteria virulence (Grant and Jones, 2009). However, ABA positively regulates tomato resistance to biotic stress by participating in the lutein (Ton et al., 2009), ascorbic acid, and glutathione cycles (Bob et al., 2007). In this experiment, endogenous ABA levels in both resistant and susceptible grape varieties initially increased, then decreased after powdery mildew infestation. A similar trend was observed in melon powdery mildew studies (Ondřej and Petr, 2023), where increased ABA levels in callus triggered ROS production, thereby positively regulating defense gene expression. Furthermore, endogenous ABA levels in resistant black spiked currant varieties infested with powdery mildew were significantly higher than in susceptible varieties, indicating ABA’s crucial role in positively regulating resistance to powdery mildew in black currant leaves. This is consistent with findings in barley (Chen et al., 2013) post-infestation with powdery mildew. Thus, ABA’s effectiveness in enhancing plant resistance may vary depending on plant species, pathogen type, and infestation mode.

GA_3_ is associated with resistance to biotic stress (Zhe et al., 2022). However, in barley (Jovaras et al., 2024) and pea (Bhosle et al., 2019), the major synthesizing genes and endogenous GA_3_ content decreased This trend aligns with our experiment, where endogenous GA_3_ content in black currant leaves declined rapidly after powdery mildew infestation and then fluctuated at lower levels. Bryan (Bryan and Michela, 2020) found that exogenous GA_3_ spraying on grapes significantly enhanced resistance to biotic stresses. In our study, the GA_3_ content in resistant varieties was significantly higher than in susceptible varieties during the uninfested period, suggesting that GA_3_ may regulate powdery mildew resistance in black currant.

Endogenous SA content increases after plants are stressed by pathogenic bacteria. For example, in tobacco (QiuFen et al., 2023) and *Arabidopsis thaliana (* Wildermuth et al., 2001), endogenous SA increased following powdery mildew infestation. SA acts as a signaling molecule to induce systemic resistance and enhance biotic stress tolerance. In octoploid strawberries, SA signaling activation significantly enhanced resistance to powdery mildew (Wildermuth et al., 2001). Similarly, exogenous SA spraying enhanced powdery mildew resistance in roses (Fazhong et al., 2022). In our experiment, endogenous SA content in all resistant varieties increased significantly after powdery mildew infestation, consistent with the response observed in other plants. Additionally, the endogenous SA content in resistant black currant varieties was higher than in susceptible ones post-infestation, further supporting the positive correlation between endogenous SA content and disease resistance. This suggests that SA may also regulate powdery mildew resistance in black currant.

### 4.4 Transcriptome analysis of blackcurrant infested with powdery mildew

Previously, genetic studies of black currant disease focused on the black currant retrovirus (BRV). Brennan’s study (Brennan et al., 2008) localized the *Ce* gene using AFLP, SSR, SNP, and transcriptome analyses. Mažeikienė (Mažeikienė et al., 2022) screened 221 and 850 BRV-related DEGs from 2dpi and 4dpi,respectively, and analyzed the phenylpropanoid biosynthesis pathways, cutin, suberine and wax biosynthesis pathways for BRV activation. However, fungal diseases have been less studied at the gene level. This study will analyze four important pathways in detail using transcriptome master data.

The phytohormone synthesis pathway is enriched in almost any crop infested with powdery mildew, such as Tibetan hull-less barley (Hongjun et al., 2018), tobacco (Rong et al., 2023) and watermelon (Vivek et al., 2022). In this experiment, many transcription factors were up- and down-regulated in the phytohormone synthesis pathway, with the highest number of gene changes in the oleoresin lactone synthesis pathway. The alterations in genes associated with the oleoresin lactone synthesis pathway in response to powdery mildew in grapes (Bhatia et al., 2021) were comparable to those observed in this study. The authors concluded that boosting this pathway could enhance resistance to powdery mildew in grapes. Several studies have shown that oleuropein lactones can enhance resistance to fungal diseases. For instance, in *Eucalyptus megacephalus* (Shae et al., 2021), the resistant varieties had significantly more active oleuropein lactone pathways than in susceptible ones post-rust infestation. In another study, bananas (Yunhao et al., 2021) reportedly improved resistance to endophytic *Bacillus subtilis* TR21 by up-regulating the oleuropein lactone synthesis pathway. The present experiment found that relevant oleoresin lactone synthesis pathway genes, like BR signal kinase, protein steroid-insensitive type 2, and xylose: xylosyltransferase TCH4, were significantly down-regulated during the infestation process. However, medication significantly up-regulated these genes, suggesting that drug control may enhance resistance to powdery mildew fungus in black currant by up-regulating the oleoresin lactone synthesis pathway. Methyl jasmonate consistently up-regulated genes related to JAZ proteins, structural domains of jasmonic acid, throughout infestation and control processes. Notably, the JAZ4 gene, derived from wild grapes, significantly enhances resistance to powdery mildew in Arabidopsis (Guofeng et al., 2019). Thus, the methyl jasmonate synthesis pathway in black currant leaves is likely related to powdery mildew resistance. Several studies have demonstrated that the PR1 gene induces a hypersensitive response in plants, leading to systemic acquired resistance and playing a crucial role in resistance to biotic stresses (Jingru et al., 2021). As shown in **Figure 5A**, PR1-related genes were quickly up-regulated following powdery mildew infestation in this experiment, suggesting that PR1 in black currant leaves initiated a hypersensitive response during the early stages of infestation, positively regulating powdery mildew resistance. Previous studies have shown that TGA, a gene in the salicylic acid synthesis pathway, enhances strawberry (Jun et al., 2020b) resistance to powdery mildew. In this experiment, TGA-related genes were up-regulated after treatment, indicating that the activation of the TGA gene and the enhancement of endogenous SA synthesis through TGA ultimately improved powdery mildew resistance.

Flavonoids, secondary metabolites with potent antioxidant and free radical scavenging properties, bolster plant resistance by preventing pathogen invasion (Rong et al., 2023). The flavonoid content in wine grape varieties typically increases following powdery mildew infestation (Huan et al., 2022), indicating that these compounds, key products of phenylpropane metabolism, are positively linked to plant disease resistance. In our experiment, differential genes were enriched in the flavonoid metabolism pathway after powdery mildew infestation in both the susceptible black currant variety, LiangYe, and tobacco (Jun et al., 2020b). In powdery mildew-infested leaves, the expression of F3H (flavanone 3-hydroxylase), DFR (dihydroflavonol reductase), C3H (5-O-(4-coumaroyl)-D-quinate 3’-monooxygenase), and CYP73A (trans-cinnamate 4-monooxygenase) was down-regulated, while ANR (anthocyanidin reductase) was up-regulated. Conversely, drug control spraying reversed this trend. A study on cassava resistance against cassava cotton mealybug (Yue et al., 2023) found a positive correlation between F3H, DFR, and resistance, suggesting that the up-regulation of F3H and DFR-related genes observed after control in our experiment positively regulates powdery mildew resistance in black currant. Previous research has shown that ANR reduces salt stress tolerance in apple by modulating osmoregulatory substances (Wang et al., 2023), explaining the up-regulation of ANR after powdery mildew infestation and its down-regulation after control in our experiment. Thus, it is hypothesized that ANR expression in black cohosh currant leaves may facilitate pathogen infestation and weaken resistance to powdery mildew.

After infestation with powdery mildew, black currant exhibited up-regulation of the 8-carboxyestosterol dehydrogenase and 8-carboxyaromannan synthase genes in the enriched monoterpene synthesis pathways. In contrast, the linalool synthase gene was differentially regulated in wheat; it was up-regulated in resistant wheat but down-regulated in susceptible wheat three hours post-infestation (Yue et al., 2023), implicating its role in pre-existing defense against wheat powdery mildew. In the current study, the susceptible wheat variety Leafy did not exhibit significant changes in the linalool synthase gene during powdery mildew infestation. However, up-regulation was induced by powdery mildew treatment. This suggests that the treatment used to control powdery mildew activates the linalool synthase gene in the monoterpene synthesis pathway, thereby contributing to powdery mildew control.

Sugars are vital carbon sources that sustain fungal survival and promote reproduction during fungal disease infestation. Pathways converting pentose and hexanedioxylic acid were enriched in comparisons of powdery mildew-infested black currant leaves and controls treated with powdery mildew spray. During infestation, genes encoding glucuronide kinase, uridine diphosphate glucose 6-dehydrogenase, and xylitol kinase were significantly down-regulated, while those encoding D-xylulose reductol and L-methylulose 2-dehydrogenase were up-regulated. The powdery mildew fungus utilized glucuronides and xylitol, metabolized by these genes, as carbon sources to sustain its activity and further infestation. Following the application of powdery mildew control, genes for glucuronide kinase and uridine diphosphate glucose 6-dehydrogenase were up-regulated. Consequently, the utilization of UDP-DD glucuronide decreased as the powdery mildew fungus was inhibited. Additionally, genes for sucrose phosphatase and polygalacturonase were up-regulated. Polygalacturonase possibly facilitated restoration of the physical and physiological structure of the leaves (Josip et al., 2023). Although polygalacturonase is known to degrade cell walls, enhancing virulence (Garima et al., 2022), fruit softening (López-Casado et al., 2023), and promoting fungal disease infestation, the up-regulated polygalacturonase gene in this study may positively regulate powdery mildew resistance (**Figure 5D**). Similar findings were reported in strawberry fruit studies, where polygalacturonase enhanced grey mold resistance by inhibiting the protein (Pingjing et al., 2024).

## 5 Conclusion

(1) Powdery mildew infection of black currant leaves damages the photosynthetic capacity of susceptible varieties more severely than that of resistant varieties.
(2) The POD, PPO, CAT, PAL, and SOD enzymes positively regulated powdery mildew resistance; thus, enhancing their activity can bolster black currant resistance to powdery mildew.
(3) Powdery mildew resistance can be achieved by elevating endogenous ABA, GA_3_, and SA content, and by inhibiting endogenous IAA during the early stage of disease susceptibility.
(4) During powdery mildew infection and control in black currant, differentially expressed genes were co-enriched in several pathways: monoterpene synthesis, pentose and hexanedioic acid conversion, flavonoid biosynthesis, and phytohormone synthesis. Notably, flavanone 3-hydroxylase (F3H) and dihydroflavonol reductase (DFR) positively regulated powdery mildew resistance, while anthocyanin reductase (ANR) and polygalacturonase (PG) negatively regulated it. Future studies should prioritize these transcription factors to regulate powdery mildew resistance in black currant.

## Data Availability

The transcriptome data has been published at NCBI as https://www.ncbi.nlm.nih.gov/bioproject/PRJNA1142740/.
